# Results from a biodiversity experiment fail to represent economic performance of semi-natural grasslands

**DOI:** 10.1038/s41467-021-22309-7

**Published:** 2021-04-09

**Authors:** Bettina Tonn, Martin Komainda, Johannes Isselstein

**Affiliations:** 1 Grassland Science, Department of Crop Sciences, University of Göttingen, Göttingen, Germany; 2 Centre of Biodiversity and Sustainable Land Use, University of Göttingen, Göttingen, Germany

**Keywords:** Agroecology, Agriculture

**Arising from** Schaub et al. *Nature Communications* 10.1038/s41467-020-14541-4 (2020)

Semi-natural grasslands are among the most diverse and valuable habitats in temperate Europe^1^ but are highly endangered by both intensification and abandonment of agricultural management^2–4^. By contrast, grass for livestock feeding is nowadays predominantly grown in relatively species-poor, intensively managed grasslands in most parts of Europe^5^. Finding ways to preserve semi-natural grassland diversity in a way that is economically viable for land managers is, therefore, a highly relevant research aim.

Based on results from a grassland biodiversity experiment, Schaub et al.^6^ recently concluded that increasing diversity in semi-natural grasslands had the same positive effect on economic revenue from livestock production as increasing fertilization and cutting frequency. We question this conclusion for two reasons, namely (i) that the biodiversity experiment used is not suited to address these management questions in real-life systems, and (ii) that basing economic revenue on quality-adjusted yields leads to unrealistic and biased results.

## Real-life systems are different

The experiment underlying the study provides a diversity gradient of 1–60 plant species, established in assemblages randomly chosen from a pool of species typical of *Arrhenatheretum* grasslands. Recently sown on fertile arable soil and maintained by weeding, this experiment is a highly artificial system that fails to meet the definition of semi-natural grasslands^7^. Four years after establishment, a management intensity gradient of one to four annual cuts and three fertilization levels was established in subplots randomly assigned to the 1–60-species plots. Data presented in this study were collected in the following year.

Intensive management was thus imposed on plant species typical of *Arrhenaterethum* meadows, a plant community characterized by two annual cuts^8^. The potential effect size of increased management intensity is thus underestimated by applying the management to a plant community not adapted to it. More importantly, it is unlikely that the species-richness of high-diversity plots could be maintained under increased management intensity over longer periods. In fact, 22% of these subplots managed at very high intensity had to be excluded for missing or insufficient yield after only two years, indicating that their species did not persist under high defoliation frequency and fertilizer levels, even when competitors were excluded by weeding.

While the discussion hardly addresses this crucial trade-off between management intensity and plant diversity, Schaub et al.^6^ do indicate that repeated resowing is likely to be necessary to maintain high diversity under increased management intensities. In contrast to permanent grasslands, whose species composition is shaped by site conditions and management, species selection in (re-)sown grasslands is a conscious choice. To be advantageous, mixtures have to show larger yields than the most productive monoculture, so-called transgressive overyielding. Transgressive overyielding is one of the reasons why mixtures, especially grass-clover mixtures, are frequently used in sown grasslands. A European-scale experiment demonstrated that four-species mixtures showed transgressive overyielding at a wide range of sites under intensive agricultural management^9,10^. Although Schaub et al.^6^ generally quantify the diversity effects in comparison to monocultures, they argue that grasslands with the high-diversity characteristic of semi-natural grasslands have benefits not only over monocultures but over low-diversity grasslands, such as the 1–8 species standard mixtures shown in Fig. 6 of their paper. However, their results fail to demonstrate that their high-diversity plots show any transgressive overyielding even over monocultures, not to speak of low-diversity mixtures. As species assemblages of the experiment are randomly drawn from the species pool, monocultures and low-diversity mixtures cannot be expected to include the most productive species or species combinations and thus cannot be used to assess transgressive overyielding. When transgressive overyielding was quantified for one- to eight-species plots of the same experiment under extensive management in 2003, it decreased with species number. While two-species mixtures showed a mean transgressive overyielding of 5%, eight-species mixtures were only 70% as productive as the corresponding best monoculture, on average^11^.

Accordingly, the experimental design fails to capture the real trade-offs faced by grassland managers, either in permanent or in sown grassland. It cannot answer if high levels of diversity and the associated biodiversity benefits can be maintained under intensive management for a longer period than just a few years. Neither can it show a productivity benefit of high-diversity grassland assemblages compared to species-poor mixtures, or even monocultures, when in practice the sown species are deliberately chosen rather than randomly drawn from a species pool. While the underlying biodiversity experiment has made valuable contributions to our fundamental understanding of plant diversity effects on ecosystem functioning, it thus cannot be used to derive direct management recommendations for managed grassland.

## Economic revenue on quality-adjusted yields leads to unrealistic results

A further bias is introduced to the study by calculating economic revenues based on ‘quality-adjusted yields.’ This assumes that biomass yields and forage quality are interchangeable in terms of livestock performance, ignoring that daily dry matter intake of ruminants is limited: Rather than being able to compensate for lower forage energy content by higher dry matter intake, ruminants decrease their dry matter intake as forage energy content declines^12^. As a consequence, forage quality rather than biomass yield is often limiting the agronomic use of semi-natural grasslands^13^. Live weight gain or milk production depends on the energy surplus after the maintenance energy requirement of the organism has been met. An increasing forage energy content, and thus daily energy intake, means that a larger proportion of feed energy is available for milk or meat production.

Based on the data for biomass yields and metabolizable energy contents provided by Schaub et al.^14^, we recalculated the milk production potential yield, considering both forage intake and the energy requirement for maintenance. We calculated daily forage intake per cow as forage dry matter intake (kg d^−^^1^) = 2.75 × net energy for lactation (MJ kg^−1^) + 0.5^12^. As only the content of metabolizable energy is included in the published dataset^14^, we estimated net energy for lactation (NEL) as NEL (MJ kg^−^^1^) = 0.6 × (1 + 0.004 × *q* −0.57) × metabolizable energy (MJ kg^−^^1^), where *q* is the quotient between metabolizable energy and gross energy (set at 18.4 MJ kg^−^^1^)^15^. We assumed a maintenance energy of 35.5 MJ d^−^^1^ NEL for a cow of 600 kg live weight, and an energy requirement of 3.284 MJ NEL to produce 1 kg of milk [15, see Supplementary Information for more details].

**Fig. 1:**
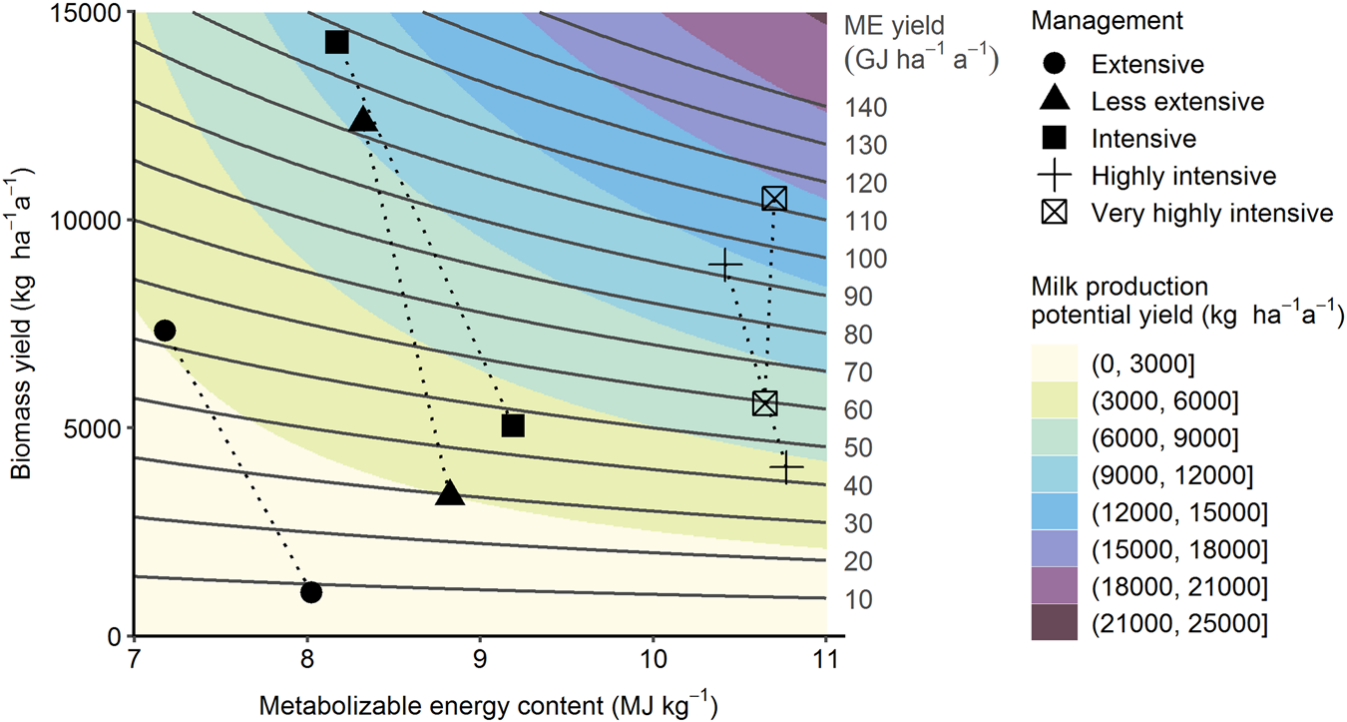
Recalculated milk production potential yield. Milk production potential yield (shading) and metabolizable energy (ME) yield (solid lines) as a function of biomass yield and forage metabolizable energy content. Symbols connected by dotted lines represent mean values of the 1-species plots (lower biomass yield) and 60-species plots (higher biomass yield) under the different management treatments as reported in ref. ^14^.

**Fig. 2:**
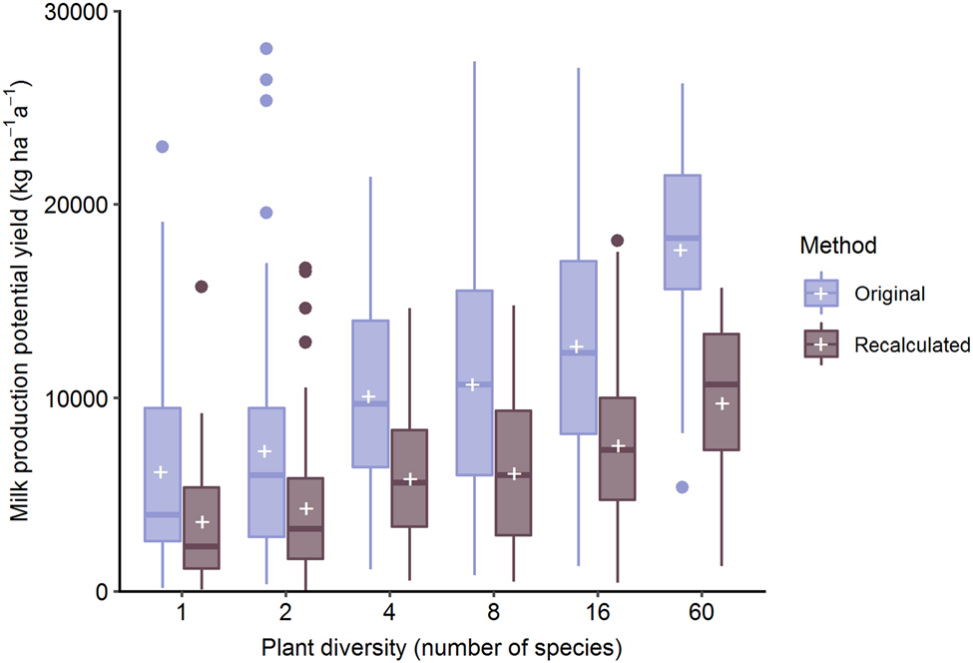
Bias in calculated milk production potential yield introduced by omitting maintenance energy requirements and daily dry matter intake capacity of dairy cows. Milk production potential yields as calculated in the original paper by Schaub et al.^6^ (original), compared to values recalculated to include maintenance energy requirements and daily dry matter intake capacity of dairy cows (‘recalculated’), as a function of plant diversity. Boxplots: center line, median; box limits, upper and lower quartiles; whiskers, 1.5× interquartile range; points, outliers; cross, arithmetic mean.

Our results show that even at constant metabolizable energy yields, milk production potential yield increases with forage metabolizable energy content (Fig. 1). Not incorporating forage intake capacity and maintenance energy requirements led Schaub et al.^6^ to overestimate milk production potential yield by 4318 kg ha^−^^1^ a^−^^1^ and revenues by 1339 € ha^−^^1^ a^−^^1^, averaged across all plots. In addition, their calculation introduces a bias milk production potential yield per plant diversity level: It was overestimated by 2592 kg ha^−^^1^ a^−^^1^ at a plant diversity of 1, but by three times that amount (7895 kg ha^−^^1^ a^−^^1^) at a plant diversity of 60 (Fig. 2).

Thus, the conclusion that less intensively managed, diverse grasslands can economically compete with intensively managed, species-poor grasslands have to be questioned on methodological grounds. Such a claim, moreover, is not without consequences for the conservation of species-rich semi-natural grasslands, as it challenges the current necessity of monetary support for farmers maintaining these grasslands. Given the great ecological value of semi-natural grasslands, developing livestock systems that decrease dependence on such support is an urgent research priority.

### Reporting summary

Further information on research design is available in the Nature Research Reporting Summary linked to this article.

## Supplementary information

Supplementary Information

Reporting Summary

## Data Availability

The data used are available at https://www.research-collection.ethz.ch/handle/20.500.11850/37410079 and in the Supplementary Information.

## Code availability

No custom code was used.
