# Supplementary material for: Results from a biodiversity experiment fail to represent economic performance of semi-natural grasslands
Source: Nat Commun. 2021 Apr 9;12:2125. doi: 10.1038/s41467-021-22309-7 (PMC8035328; doi:10.1038/s41467-021-22309-7)
Supplement: Supplementary file 1 — Supplementary Information [file 41467_2021_22309_MOESM1_ESM.pdf]

SUPPLEMENTARY INFORMATION

Tonn et al.: Results from a biodiversity experiment fail to represent economic performance of semi-natural grasslands
published in Nature Communications

Supplementary Methods

Calculation of net energy for lactation from metabolizable energy data

Different measures can be used by animal nutritionists to evaluate forage energy content. In contrast to gross energy, the energy released by complete combustion, metabolizable energy (ME) measures the energy directly available for the animal metabolism and excludes energy lost to the animal via faeces and urine. Net energy is the part of ME that is available for processes such as maintenance, weight gain or lactation. It depends on the conversion efficiency of these processes, which may differ from one process to another. Net energy lactation (NEL), which is based on the conversion efficiency of ME for lactation, is commonly used to calculate maintenance and lactation energy requirements of dairy cows. Schaub et al. calculated both ME and NEL from data of forage chemical composition and fermentability. They used NEL to calculate maintenance and lactation energy requirements. The published data set [1] contains data for ME but not NEL. To reproduce the calculations of Schaub et al., we thus had to calculate NEL from ME as

NEL (MJ kg^{-1}) = e × ME (MJ kg^{-1}),

where e is the conversion efficiency of ME for lactation. This conversion efficiency increases with increasing forage metabolisability (q), *i.e.*, an increasing quotient between metabolizable energy and gross energy, as follows [2]:

$e = 0.6 \times (1 + 0.004 \times q - 0.57)$

While metabolisability can vary strongly between different grassland-based forages, their gross energy contents are far more similar. Gross energy content of a forage can be calculated as follows [2]:

gross energy (kJ kg^{-1}) = 23.9 CP (g kg^{-1}) + 39.8 CL (g kg^{-1}) + 20.1 CF (g kg^{-1}) + 17,5 NfE (g kg^{-1})

where CP is crude protein content, CL is crude fat content, CF is crude fibre content and NfE is nitrogen-free extract content.

We applied this formula to typical forage composition data [3], covering all values provided for grass-dominated and forb- and legume-dominated meadows. The resulting gross energy content ranged from 17.9 to 18.9 MJ kg^{-1}, with a mean value of 18.4 MJ kg^{-1}. Accordingly, we chose the value of 18.4 MJ kg^{-1} to calculate q from the ME values presented in [1].

Calculation of daily forage intake per cow

The amount of dry matter that can be consumed by a dairy cow increases with increasing digestibility of the forage, as this increases rumen passage rates. We thus used a formula that calculates daily forage dry matter intake as a function of the content of NEL [4]:

forage dry matter intake (kg d^{-1}) = 2.75 × NEL (MJ kg^{-1}) + 0.5.

Calculation of energy requirements

The energy requirement for maintenance is a function of metabolic body weight, *i.e.*, body weight to the power of 0.75. For dairy cows, it has been determined as [5]:

NEL requirement (MJ d^{-1}) = 0.293 * live weight (kg) $^{0.75}$

We assumed a live weight of 600 kg per cow, which is a typical value for European dairy cows.

The energy required to produce one kilogram of milk depends on the concentration of fat (F) and protein (P) in the milk, as follows [5]:

NEL requirement (MJ kg^{-1}) = 0.038 * F (g kg^{-1}) + 0.021 P (g kg^{-1}) + 1.05

For better comparability between milk yields when milk composition differs, milk yields are usually expressed as energy corrected milk, with a fat content of 40 g kg^{-1} and a protein content of 34 g kg^{-1}. Accordingly, we used these values for fat and protein content in our calculation, yielding an NEL requirement of 3.284 MJ for 1 kg of milk.

Supplementary References

1. Schaub, M. et al. Data: forage quality and biomass yield of the Management Experiment set up within the Jena Experiment. *ETH Zur. Res. Collect* https://doi.org/10.3929/ethz-b-000374100 (2019).
2. Stangl, G.I. in *Tierernährung: Leitfaden für Studium, Beratung und Praxis.* Ch. 4 (DLG-Verlag, Frankfurt am Main, 2011).
3. DLG–Deutsche Landwirtschaftsgesellschaft. *Datenbank Futtermittel.* http://datenbank.futtermittel.net (2020).
4. Jans, F., Kessler, J., Münger, A. & Schlegel, P. in *Fütterungsempfehlungen für Wiederkäuer (Grünes Buch)* Ch. 7 (Agroscope, Posieux, 2015).
5. Schwarz, F.J. in *Tierernährung: Leitfaden für Studium, Beratung und Praxis.* Ch. 7 (DLG-Verlag, Frankfurt am Main, 2011).
