# Supplementary material for: Results from a biodiversity experiment fail to represent economic performance of semi-natural grasslands
Source: Nat Commun. 2021 Apr 9;12:2125. doi: 10.1038/s41467-021-22309-7 (PMC8035328; doi:10.1038/s41467-021-22309-7)
Supplement: Supplementary file 2 — Reporting Summary [file 41467_2021_22309_MOESM2_ESM.pdf]

nature research

Corresponding author(s): Bettina Tonn

Last updated by author(s): Feb 17, 2021

Reporting Summary

Nature Research wishes to improve the reproducibility of the work that we publish. This form provides structure for consistency and transparency in reporting. For further information on Nature Research policies, see Authors & Referees and the Editorial Policy Checklist .

Statistics

For all statistical analyses, confirm that the following items are present in the figure legend, table legend, main text, or Methods section.

n/a	Confirmed	
x	☐	The exact sample size (n) for each experimental group/condition, given as a discrete number and unit of measurement
x	☐	A statement on whether measurements were taken from distinct samples or whether the same sample was measured repeatedly
x	☐	The statistical test(s) used AND whether they are one- or two-sided<br>*Only common tests should be described solely by name; describe more complex techniques in the Methods section.*
x	☐	A description of all covariates tested
x	☐	A description of any assumptions or corrections, such as tests of normality and adjustment for multiple comparisons
x	☐	A full description of the statistical parameters including central tendency (e.g. means) or other basic estimates (e.g. regression coefficient) AND variation (e.g. standard deviation) or associated estimates of uncertainty (e.g. confidence intervals)
x	☐	For null hypothesis testing, the test statistic (e.g. F, t, r) with confidence intervals, effect sizes, degrees of freedom and P value noted<br>*Give P values as exact values whenever suitable.*
x	☐	For Bayesian analysis, information on the choice of priors and Markov chain Monte Carlo settings
x	☐	For hierarchical and complex designs, identification of the appropriate level for tests and full reporting of outcomes
x	☐	Estimates of effect sizes (e.g. Cohen's d, Pearson's r), indicating how they were calculated

Our web collection on statistics for biologists contains articles on many of the points above.

Software and code

Policy information about availability of computer code

Data collection	Data were retrieved from:<br>Schaub, M. et al. Data: forage quality and biomass yield of the Management Experiment set up within the Jena Experiment. ETH Zur. Res. Collect https://doi.org/10.3929/ethz-b-000374100 (2019).
Data analysis	Data were analysed with R version 3.6.0 (R Core Team 2019. R: A language and environment for statistical computing. R Foundation for Statistical Computing, Vienna, Austria. URL https://www.R-project.org/.)

For manuscripts utilizing custom algorithms or software that are central to the research but not yet described in published literature, software must be made available to editors/reviewers. We strongly encourage code deposition in a community repository (e.g. GitHub). See the Nature Research guidelines for submitting code & software for further information.

Data

Policy information about availability of data

All manuscripts must include a data availability statement. This statement should provide the following information, where applicable:

- Accession codes, unique identifiers, or web links for publicly available datasets
- A list of figures that have associated raw data
- A description of any restrictions on data availability

The data used for re-calculation is available at https://www.research-collection.ethz.ch/handle/20.500.11850/374100 79.

Field-specific reporting

Please select the one below that is the best fit for your research. If you are not sure, read the appropriate sections before making your selection.

Ecological, evolutionary & environmental sciences study design

All studies must disclose on these points even when the disclosure is negative.

Study description

We used the data set presented by Schaub et al. (2020) and available as Schaub et al. (2019). We recalculated milk production potential yield by including forage intake capacity and energy requirements for maintenance of dairy cows. We based this calculation on the data of metabolisable energy content and biomass yield reported in Schaub et al. (2019), and on standard values from the literature as presented in our manuscript text.
As we exclusively used the data collected by Schaub et al. (2020), we refer to the Reporting Summary of that paper for the details below.

References:

Schaub, M. et al. Plant diversity effects on forage quality, yield and revenues of semi-natural grasslands. Nature Comm. 11, 768 (2020)
Schaub, M. et al. Data: forage quality and biomass yield of the Management Experiment set up within the Jena Experiment. ETH Zur. Res. Collect https://doi.org/10.3929/ethz-b-000374100 (2019).

Research sample

See Reporting Summary of Schaub et al. (2020)

Sampling strategy

See Reporting Summary of Schaub et al. (2020)

Data collection

See Reporting Summary of Schaub et al. (2020)

Timing and spatial scale

See Reporting Summary of Schaub et al. (2020)

Data exclusions

See Reporting Summary of Schaub et al. (2020)
In our own calculations, we used the full available data set.

Reproducibility

See Reporting Summary of Schaub et al. (2020)

Randomization

See Reporting Summary of Schaub et al. (2020)

Blinding

See Reporting Summary of Schaub et al. (2020)

Did the study involve field work? [x] Yes [ ] No

Field work, collection and transport

Field conditions

See Reporting Summary of Schaub et al. (2020)

Location

See Reporting Summary of Schaub et al. (2020)

Access and import/export

See Reporting Summary of Schaub et al. (2020)

Disturbance

See Reporting Summary of Schaub et al. (2020)

Reporting for specific materials, systems and methods

We require information from authors about some types of materials, experimental systems and methods used in many studies. Here, indicate whether each material, system or method listed is relevant to your study. If you are not sure if a list item applies to your research, read the appropriate section before selecting a response.

Materials & experimental systems

n/a	Involved in the study
[x]	[ ] Antibodies
[x]	[ ] Eukaryotic cell lines
[x]	[ ] Palaeontology
[x]	[ ] Animals and other organisms
[x]	[ ] Human research participants
[x]	[ ] Clinical data

Methods

n/a	Involved in the study
[x]	[ ] ChIP-seq
[x]	[ ] Flow cytometry
[x]	[ ] MRI-based neuroimaging
